# Unraveling Macrophage Heterogeneity in Erythroblastic Islands

**DOI:** 10.3389/fimmu.2017.01140

**Published:** 2017-09-20

**Authors:** Katie Giger Seu, Julien Papoin, Rose Fessler, Jimmy Hom, Gang Huang, Narla Mohandas, Lionel Blanc, Theodosia A. Kalfa

**Affiliations:** ^1^Cancer and Blood Diseases Institute, Cincinnati Children’s Hospital Medical Center, University of Cincinnati College of Medicine, Cincinnati, OH, United States; ^2^Laboratory of Developmental Erythropoiesis, Center for Autoimmune and Musculoskeletal Diseases, The Feinstein Institute for Medical Research, Manhasset, NY, United States; ^3^Red Cell Physiology Laboratory, Lindsey F Kimball Research Institute, New York Blood Center, New York, NY, United States; ^4^Department of Molecular Medicine and Pediatrics, Hofstra-Northwell School of Medicine, Hempstead, NY, United States

**Keywords:** erythropoiesis, erythroblastic islands, macrophages, imaging flow cytometry, CD11b, VCAM-1, CD169, CD163

## Abstract

Mammalian erythropoiesis occurs within erythroblastic islands (EBIs), niches where maturing erythroblasts interact closely with a central macrophage. While it is generally accepted that EBI macrophages play an important role in erythropoiesis, thorough investigation of the mechanisms by which they support erythropoiesis is limited largely by inability to identify and isolate the specific macrophage sub-population that constitute the EBI. Early studies utilized immunohistochemistry or immunofluorescence to study EBI morphology and structure, while more recent efforts have used flow cytometry for high-throughput quantitative characterization of EBIs and their central macrophages. However, these approaches based on the expectation that EBI macrophages are a homogeneous population (F4/80^+^/CD169^+^/VCAM-1^+^ for example) provide an incomplete picture and potentially overlook critical information about the nature and biology of the islands and their central macrophages. Here, we present a novel method for analysis of EBI macrophages from hematopoietic tissues of mice and rats using multispectral imaging flow cytometry (IFC), which combines the high-throughput advantage of flow cytometry with the morphological and fluorescence features derived from microscopy. This method provides both quantitative analysis of EBIs, as well as structural and morphological details of the central macrophages and associated cells. Importantly, the images, combined with quantitative software features, can be used to evaluate co-expression of phenotypic markers which is crucial since some antigens used to identify macrophages (e.g., F4/80 and CD11b) can be expressed on non-erythroid cells associated with the islands instead of, or in addition to the central macrophage itself. We have used this method to analyze native EBIs from different hematopoietic tissues and evaluated the expression of several markers that have been previously reported to be expressed on EBI macrophages. We found that VCAM-1, F4/80, and CD169 are expressed heterogeneously by the central macrophages within the EBIs, while CD11b, although abundantly expressed by cells within the islands, is not expressed on the EBI macrophages. Moreover, differences in the phenotype of EBIs in rats compared to mice point to potential functional differences between these species. These data demonstrate the usefulness of IFC in analysis and characterization of EBIs and more importantly in exploring the heterogeneity and plasticity of EBI macrophages.

## Introduction

Erythropoiesis is the most demanding aspect of hematopoiesis in terms of number of cells that need to be produced each day. An adult human throughout his life span produces approximately 2.5 million red blood cells per second (1) and an adult mouse 7,000 red blood cells per second under steady-state condition.^1^ During mammalian development, erythropoiesis occurs in three waves; primitive, fetal definitive, and adult definitive (4). Each of these waves is regulated both temporally and spatially. After the primitive wave occurs in the yolk sac, erythropoiesis migrates to the fetal liver (FL) and finally to the bone marrow (BM) (4). In the mouse, the BM cannot expand significantly to mount stress erythropoiesis, in which case the spleen (SP) becomes the major erythropoietic organ (5).

Erythropoiesis begins with the commitment of a hematopoietic stem cell (HSC) into the megakaryocyte-erythroid lineage by differentiation to the megakaryocyte-erythroid progenitor. Within this population, the burst-forming unit-erythroid and the more mature colony-forming unit-erythroid (CFU-E) progenitors are exclusively committed to the erythroid lineage and generate the erythropoietic precursors, which are identified based on their morphology as proerythroblasts, basophilic, polychromatophilic, and orthochromatic erythroblasts (6). The erythroblast phase or terminal erythropoiesis concludes after four to five cell divisions with the enucleation of the orthochromatic erythroblast which generates a pyrenocyte, containing the nucleus, and a reticulocyte that further matures into a red blood cell (7–9).

From the CFU-E to the nascent reticulocyte, differentiating erythroid progenitors and precursors are in close contact with a central macrophage. Together, they form an anatomical structure, that was first defined in 1958 as “the functional unit of the bone marrow” by Marcel Bessis who designated these structures erythroblastic islands (EBIs) (10). Observed at first in the rat BM, EBIs have been recognized as niches for erythropoiesis, where positive and negative regulation of terminal erythroid differentiation is organized and coordinated. They have been observed in different erythropoietic organs, such as the FL, the BM and the SP of diverse mammalian species (11, 12), including human BM (13). The interactions of erythroblasts with the central macrophage and the extracellular matrix within the island are critical for optimal proliferation, survival, differentiation, and efficient enucleation (14, 15). Indeed, the central macrophage is responsible for proper disposal of the pyrenocyte once extruded, and some recent studies demonstrated that the macrophage can also provide iron for hemoglobin synthesis and growth signals to regulate erythroblast proliferation and differentiation (16, 17), a speculation originally made by Marcel Bessis in 1960s.

Although these studies have highlighted a functional role for the central macrophage, the identity of the specific phenotype of the EBI macrophage remains to be defined. One major stumbling block in the characterization of these erythropoietic niches is the lack of specific phenotypic markers to precisely discriminate an island macrophage from other resident/stromal macrophages. The F4/80 antigen has historically been used to identify EBI macrophages in murine tissues; however, F4/80 is widely expressed on eosinophils, monocytes and many, although not all, tissue resident macrophages including liver (Kupffer cells), splenic red pulp but not marginal zone, adrenal glands, and microglia (18). Additional markers such as CD169 in the mouse (19), and CD163 in the rat (20), have been identified using immunofluorescence and flow cytometry approaches. Due to the close interaction between erythroblasts and the central macrophage, critical adhesion molecules such as erythroblast macrophage protein (EMP) and vascular cell adhesion molecule-1 (VCAM-1) have also been implicated as surface markers of the central macrophage but they are also expressed on a variety of other cell types (21, 22). Flow cytometry studies recently highlighted CD11b, a monocyte marker, as a potential surface marker for the central macrophage in the mouse BM and SP (23).

In this study, we present a novel method for analysis and characterization of EBI macrophages from hematopoietic tissues using multispectral imaging flow cytometry (IFC), which combines the high-throughput advantage of flow cytometry with the morphology and fluorescence details obtained from microscopy. Using this method to study EBIs recovered directly from tissues, we are able to perform in an automated matter non-biased evaluation of structural and morphological characterization of the central macrophage and surrounding erythroblasts. Using CD11b, VCAM-1, F4/80, CD163, and CD169 as potential markers for the central macrophage, and Ter119 or Glycophorin A and CD71 as markers for erythroblasts, we analyzed these structures in mouse and rat hematopoietic tissues. While we confirmed expression of CD169 and VCAM-1 on the F4/80^+^ central macrophage of EBIs in the mouse, we also identified a population of VCAM-1^+^/F4/80^−^ cells associated with erythroblasts in clusters resembling EBIs. CD163 was detected in the EBI macrophages in rats but not in mice. Most notably, CD11b was detectable as a membrane marker in other cells present in EBIs but not in the central macrophage. These findings suggest that IFC could potentially serve as a comprehensive method for the analysis and characterization of EBIs and for unraveling the heterogeneity of EBI macrophages within the same tissue and between different hematopoietic tissues.

## Materials and Methods

C57Bl/6 mice 4–6 months of age were used because few EBIs are recovered from BM of mice less than 2 months of age. Sprague-Dawley rats used were between 3 and 5 months of age. All mouse and rat protocols were approved by the Institutional Animal Care and Use Committees of Cincinnati Children’s Hospital Medical Center, Cincinnati, OH, USA, and the Feinstein Institute for Medical Research, Manhasset, NY, USA.

### Preparation of EBI Samples from BM

Tibiae and femurs were harvested from C57Bl/6 mice and were flushed with a 25 G needle attached to a syringe containing IMDM with 20% FBS, 1 mM CaCl_2_, and 1 mM MgCl_2_ (“EBI buffer”). The BM cells and cell aggregates in suspension were gently pipetted through a 1 mL pipette to break up large pieces of tissue, filtered through a 70 µm cell strainer, and then counted and fixed with 4% PFA for 20 min at room temperature.

### Induction of Stress Erythropoiesis

Stress erythropoiesis was induced in C57BL/6 mice by phlebotomy removing 500 µL whole blood followed by fluid replacement intraperitoneally with normal saline. After 4 days, the mice were euthanized and the SPs were removed and crushed through a 70 µm filter into EBI buffer; cell count was determined and the cells were fixed with 4% PFA as above.

### Timed Pregnancies

Mice were paired for 24 h. The following morning, considered as day E0.5, females were pulled and housed in separate cages. On embryonic day E13.5, pregnant females were euthanized and embryos were collected. The FLs were harvested, gently dissociated by pipetting, and filtered through a 70 µm cell strainer; cell count was determined and the cells were fixed with 4% PFA as above.

### IFC

Fixed EBI preparations from BM (BM), SP, or FL were used within 24 h of preparation. A volume containing 10^6^ single cells was used from each sample. The cell suspensions were blocked with anti-FcR (BDBioscience) for 20 min at room temp. Samples were then centrifuged and re-suspended in FACS buffer (PBS + 0.5% BSA) containing the applicable fluorescently conjugated antibodies and Hoechst 34580 (Invitrogen). Antibodies used were anti-F4/80-AF647, anti-CD169-FITC, and PE, anti-CD106-AF488 (Biolegend); anti-Ter119-FITC, anti-CD71-PE and BV421, anti-CD11b-PE, and APC (BDBiosciences); anti-CD163-PE (R&Dsystems), anti-F4/80-AF488 (Invitrogen), and anti-CD169-FITC (AbDSerotec). Samples were covered and incubated on a shaker at 37°C with gentle mixing for 1 h and then washed twice with FACS buffer and re-suspended in the same for analysis on the ImageStream^X^ (EMD Millipore). Data was collected using a 60× objective and the classifier Area BF lower limit set to 50. The Similarity feature is the log transformed Pearson’s Correlation Coefficient and compares two images within a “masked” region, measuring the degree to which they are linearly correlated. The Circularity feature measures the degree that the “mask” of an image deviates from a circle, as expressed by the average distance of the image boundary from its center divided by the variation of this distance. The closer the object to a circle, the smaller the variation, and therefore, the feature value will be high.

### Flow Cytometry

Cells were harvested from the BM, SP, and FL of a WT mouse, stained in FACS buffer with FVD780 (eBioscience), F4/80-AF647, CD169-PE, and VCAM-AF488 (Biolegend), and analyzed on a FACSCanto flow cytometer (BD). The data were analyzed using FlowJo (FlowJo LLC, Ashland, OR, USA).

### Immunofluorescence

Fixed BM EBI suspensions prepared as described above were cytospun onto a poly-lysine-coated cover glass at 400rpm followed by additional fixation with 4%PFA for 20 min. Coverslips were washed and then incubated with antibodies overnight at 4°C in PBS + 2% BSA + 2% FBS. After washing, coverslips were incubated with DAPI in PBS + 0.1% Triton-X-100 for 10 min, washed and mounted using Fluoromount-G (SouthernBiotech).

### Statistics

Statistical analysis was performed using KaleidaGraph software v.4.1.3 (Synergy Software). Comparison between two groups with distribution of values that appeared asymmetric indicating non-normality was performed using the Wilcoxon rank-sum test that makes no assumptions about the underlying probability distributions. For each set of measurements, we report the mean and the median for each group and the two-tailed *p*-value according to the Wilcoxon rank-sum test. Significance was set at *p* < 0.05.

## Results

### Flow Cytometric Analysis of Hematopoietic Tissues

**Figure 1:**
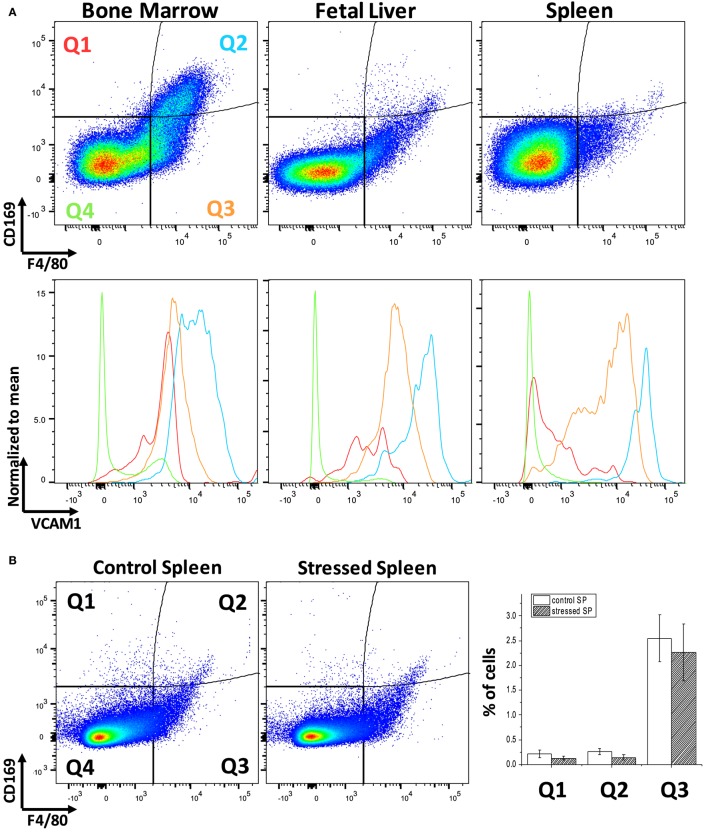
Flow cytometric analysis of F4/80, CD169, and vascular cell adhesion molecule-1 (VCAM-1) expression levels within the bone marrow (BM), fetal liver (FL), and spleen (SP) of C57Bl/6 mice. **(A)** The BM contains the largest double positive for F4/80 and CD169 population and demonstrates a high level of VCAM-1 expression. VCAM-1 expression is plotted in the lower graphs in red (Q1: F4/80^−^;CD169^+^), light blue (Q2: F4/80^+^;CD169^+^), orange (Q3: F4/80^+^;CD169^−^), or green (Q2: F4/80^−^;CD169^−^). Although the FL and stressed SP have lower amounts of F4/80^+^;CD169^+^, these double positive cells at the Q2 quadrant still express VCAM-1 highly. **(B)** Expression of CD169 and F4/80 in the spleen does not change significantly after induction of stress erythropoiesis.

Erythroblastic island macrophages have been most well characterized in BM of mammals (24) while the macrophages in EBIs in FL or in the mouse SP after induction of stress erythropoiesis have received less attention. When we compared these hematopoietic tissues in mice by flow cytometry, we found a variable expression of the known BM EBI macrophage markers: the classic murine macrophage marker F4/80, the sialoadhesin CD169, and VCAM-1 (Figure 1A). The BM contains up to 10% of cells that are double positive for F4/80 and CD169 and, also, demonstrate a high level of VCAM-1 expression. FL and stressed SP show relatively low CD169 expression; nevertheless, the F4/80^+^;CD169^+^ cells, as well as the F4/80^+^/CD169^−^ cells, still express VCAM-1 at high levels. Of note, this low expression of CD169 in the FL F4/80^+^ cells is in agreement to a study published by Siamon Gordon’s team in 1991 where sialoadhesin was not detected in FL macrophages in contrast to stromal macrophages in adult BM (19). F4/80 and CD169 expressions in splenocytes were not significantly affected by induction of stress erythropoiesis (Figure 1B). This data indicates a variable degree of expression of the known EBI macrophage markers between hematopoietic tissues and even within the same tissue. Therefore, to address the heterogeneity of this population, we sought an unbiased assay for phenotypic characterization of the EBI macrophages based on their defining characteristic: participating in EBI formation.

### Analyzing Intact EBIs with IFC

**Figure 2:**
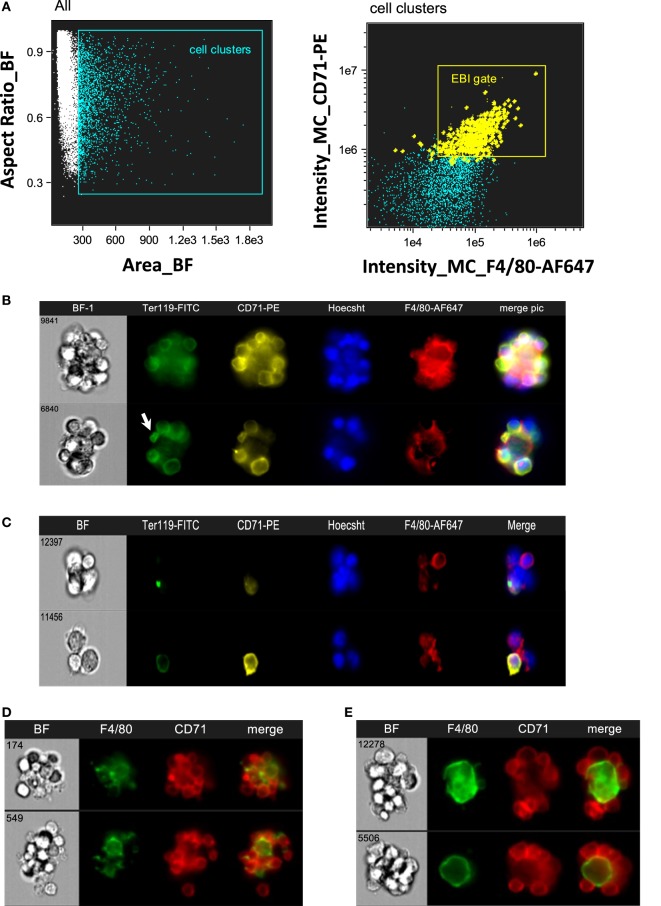
Gating strategy of erythroblastic islands (EBIs) for analysis by imaging flow cytometry (IFC). **(A)** Left panel: multiplets (cell clusters) characterized by their large area in brightfield (BF) are gated. Right panel: the cell clusters are then plotted as per intensity of F4/80 and of CD71 stain. The gate containing clusters high in fluorescence intensity for both F4/80 and CD71 is enriched in EBIs (gate). Manual inspection of the cell clusters in the EBI gate allows confirmation of the events (tagged yellow) that clearly have the structure of an EBI. Some EBIs are located outside of the EBI gate but with decreased frequency. **(B)** Examples of EBIs (representative of the yellow-marked events within the EBI gate) characterized by a central F4/80^+^ macrophage surrounded by CD71^+^ and/or Ter119^+^ erythroblasts. An anucleate reticulocyte (arrow) is occasionally seen, still in contact with the macrophage. **(C)** Examples of cell clusters, which although within the EBI gate (double positive for F4/80 and CD71), do not have the classic appearance of an intact EBI. These events would have been included in the analysis by “blind” flow cytometry, potentially leading to misguided conclusions about EBI macrophage markers. **(D)** Representative examples of EBIs isolated from mouse spleen after development of stress erythropoiesis (4 days post-induction of anemia with phlebotomy of 500 µL of blood). **(E)** Representative examples of EBIs isolated from mouse fetal livers (E13.5).

Intact EBIs were obtained by flushing BM with media containing divalent ions to preserve integrin-mediated intercellular interactions, followed immediately by fixation, preserving the structure of the native islands prior to staining and analysis on the ImageStreamX. In the analysis software, larger cell clusters are first gated on the basis of their area in the brightfield channel and then evaluated for expression of the erythroid marker CD71 and macrophage marker F4/80 (Figure 2A). The population within the double positive gate (EBI gate) is markedly enriched in EBIs, characterized by the presence of a central F4/80^+^ macrophage surrounded by several (for the purposes of this study, 3 or more) CD71^+^ erythroblasts (Figure 2B). Consistent with previous descriptions of EBIs, the participating cells often overlap considerably, indicating likely multiple intercellular interactions; extensions from the macrophage can also be seen between the associated erythroblasts. Of note, examination of the events within this population by direct observation of each cluster on IFC is necessary since the CD71^+^/F4/80^+^ double positive gate does not contain solely EBIs, but is “contaminated” with loose and/or non-specific cell clusters that do not fit the criteria of an EBI (Figure 2C). “Blind” flow cytometry could not exclude such events, potentially leading to misguided conclusions about EBI macrophage markers. True EBIs (containing a central F4/80^+^ macrophage and at least three CD71^+^ erythroblasts) comprised as low as 20–50% of the CD71^+^/F4/80^+^ double positive population. This percentage is in agreement with previous calculations by Crocker and Gordon, who concluded, after enrichment of mouse BM cell clusters by gravity sedimentation, that only ~7% of BM cells were in clusters, and only 40% of those contained erythroblasts (23). These observations underscore the advantage of IFC versus regular flow cytometry for evaluation of EBI characteristics.

The same analysis was performed with SPs from mice that had been phlebotomized to induce stress erythropoiesis (Figure 2D), as well as with mouse FL s. In FL s, EBIs with a central F4/80^+^ macrophage were identified with decreased frequency. Examination of the cell clusters outside of the F4/80^+^/CD71^+^ gate showed many island-like structures of CD71^+^ cells that lacked F4/80 positivity. Of the F4/80^+^ EBIs, one notable difference was the shape of the macrophages in FL s. While macrophages in the BM EBIs (and stressed SP EBIs) had typically an irregular appearance with many elongated extensions reaching throughout the cell cluster, in contrast, FL EBIs often had rather round, perhaps less mature, macrophages serving as the nurse-cell for erythroblasts (Figure 2E). Using the analysis software, this qualitative finding could be quantitated by the circularity feature which measures how much a staining pattern deviates from a circle. It is calculated by dividing the average distance of the stain boundary to the center by the variation of this distance. The closer an object is to a circle, the smaller the variation and thus the higher the value of the circularity feature. The median value for circularity of the FL EBI macrophages at 8.78 was significantly higher than that of the BM macrophages at 3.61, confirming the initial impression that the FL macrophages are relatively round (*p* < 0.0001). Macrophages from stressed SPs were similar in shape to the BM macrophages (median of 3.3).

### Assessment of EBI Macrophages by IFC

With a method of collecting and analyzing EBIs and their central macrophages established, we set out to validate a number of cell surface receptors that have been previously relied upon to identify the EBI macrophages.

#### CD11b

**Figure 3:**
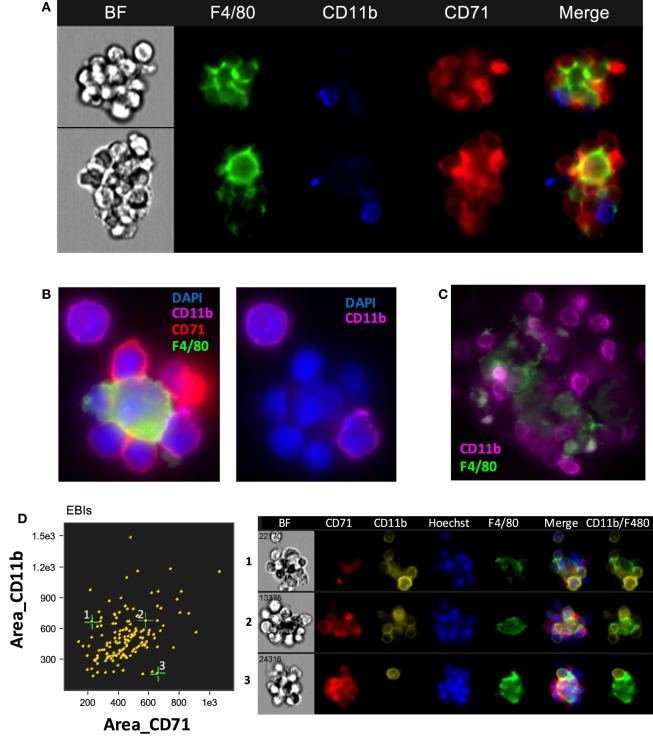
The central macrophage in erythroblastic islands (EBIs) is not CD11b^+^; however, other CD11b^+^ cells may participate in the EBI structure. **(A)** Mouse bone marrow EBIs stained with fluorescently conjugated antibodies against CD71, CD11b, and F4/80 and analyzed by imaging flow cytometry (IFC) indicate that CD11b positivity in EBIs is due to CD11b^+^ cells peripherally attached to the EBI macrophage and/or the erythroblasts; the central macrophage is not CD11b^+^. **(B)** A representative EBI imaged by immunofluorescence microscopy also showing that the F4/80^+^ central macrophage is not stained by anti-CD11b, while a cell attached to the macrophage and another one in close proximity are CD11b^+^. **(C)** Another example showing that F4/80^+^ cells in mouse bone clusters are not CD11b-bright, in contrast to adjacent cells that have a clear membranous stain for CD11b. **(D)** IFC analysis of mouse bone marrow EBIs, stained for F4/80, CD71, and CD11b indicates that CD11b^+^ cells are a consistent component of the EBI.

Jacobsen et al recently used CD11b among other markers to identify EBI macrophages by flow cytometry (25). However, our analysis using IFC showed that CD11b is not detected on the F4/80 + central macrophages; it is rather a marker of other cells participating in the EBI formation (Figure 3A). This finding is in agreement to a previous report by Crocker and Gordon which demonstrated the central macrophages of EBIs to be negative for Mac-1/CD11b (23). Of interest, the majority (up to 80%) of EBIs analyzed (520 EBIs total from 4 independent experiments with BM samples) contained at least one or more CD11b^+^ cells associated with the island, likely monocytes and neutrophils as observed by Crocker and Gordon (23). We confirmed our IFC findings with immunofluorescence microscopy of fixed BM clusters (Figures 3B,C). The ratio of EBs to CD11b^+^ cells at the islands varied, ranging from islands with only one CD11b^+^ cell among the EBs to clusters in which the CD11b^+^ cells equaled or outnumbered the EBs (Figure 3D). EBIs from FLs and stressed SPs, on the other hand, contained very few, if any, CD11b^+^ cells despite the presence of CD11b^+^ single cells in both the SP and FL (data not shown).

#### VCAM-1

**Figure 4:**
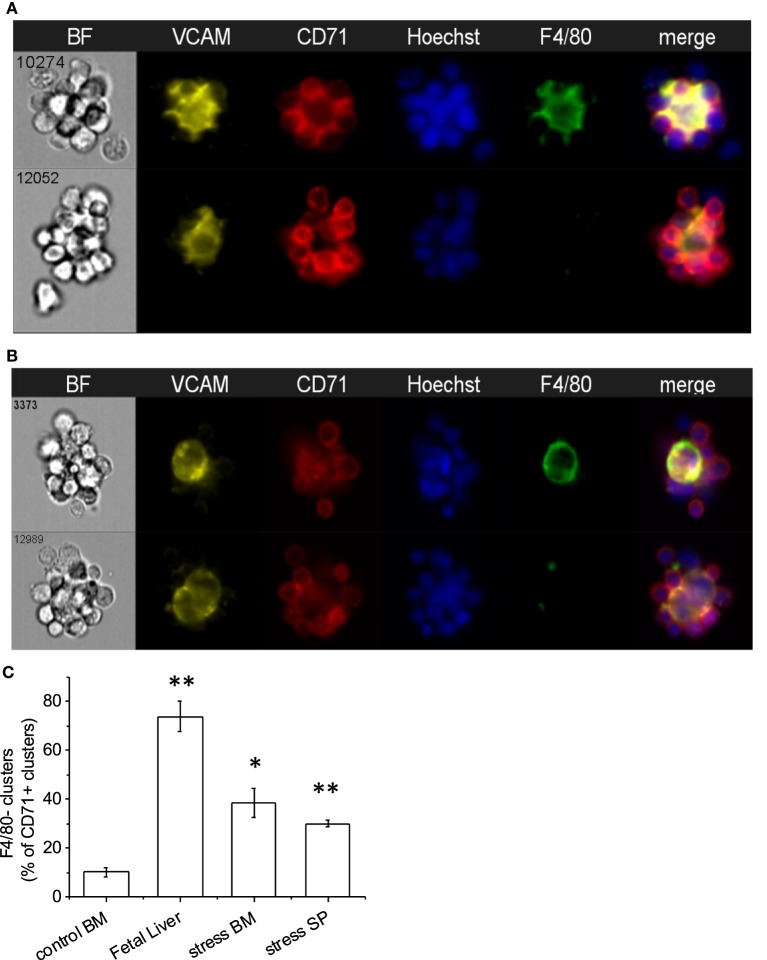
Central macrophages are VCAM-1^+^. **(A)** All of the erythroblastic island (EBI) macrophages in the mouse bone marrow are positive for vascular cell adhesion molecule-1 (VCAM-1) (top), while there are a few additional clusters of erythroblasts surrounding VCAM-1^+^;F4/80^lo^ macrophages (bottom). **(B)** Similarly, in the mouse fetal liver EBIs, all F4/80^+^ central macrophages are also VCAM-1^+^ (top). However, the clusters containing CD71^+^ erythroblasts around a central VCAM-1^+^ cell with low or intermediate F4/80 staining in the fetal liver are much more frequent than in the bone marrow (bottom). **(C)** CD71^+^ clusters with VCAM-1^+^;F4/80^lo^ macrophages are significantly increased in the fetal liver (74 ± 6%) compared to steady-state bone marrow (10 ± 2%). A smaller but significant increase is seen in the spleen (30 ± 2%) and bone marrow (39 ± 8%) after induction of stress. Data shown are mean ± SE, *p ≤ 0.05, **p ≤ 0.001.

As expected, VCAM-1, the complementary receptor for erythroblast integrin α4β1, was expressed on every F4/80^+^ EBI central macrophage in all three murine hematopoietic tissues (Figures 4A,B). A surprising finding was that there were several clusters resembling EBIs in brightfield, where CD71^+^ cells surrounded a central VCAM-1^+^ cell that, however, lacked appreciable F4/80 staining. The presence of these clusters varied in the BM samples between 5 and 20% of CD71^+^ clusters, while it increased in the BM and SPs of mice induced to develop stress erythropoiesis to up to 30–40% of total CD71^+^ clusters (Figure 4C). In addition, the presence of these F4/80^−^ clusters was quite prominent in the FL samples (between 50 and 75% of total CD71^+^ clusters) (Figure 4C). These clusters likely account for the many island-like structures of CD71^+^ cells in the FL specimens, which we observed that lack F4/80 positivity, as noted above.

#### CD169

**Figure 5:**
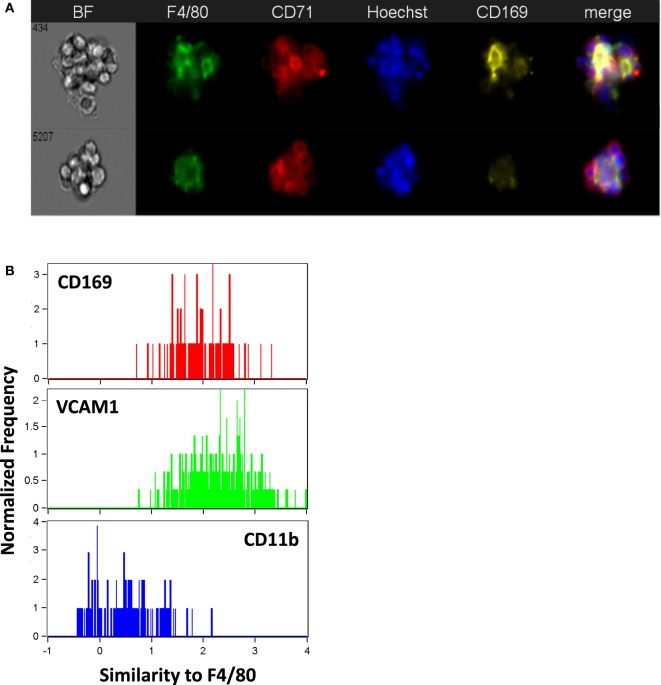
The majority of mouse bone marrow erythroblastic island (EBI) macrophages are CD169 + although the staining pattern is heterogeneous. **(A)** By imaging flow cytometry, some F4/80^+^ EBI macrophages appear CD169^+^ (top), but many do not exhibit clear staining (bottom). Staining was suboptimal with all anti-mouse CD169 antibodies tested and positivity was determined by mean pixel value since a clear image was not always observed. **(B)** The similarity feature was used to compare the staining pattern of the CD169 to that of F4/80. The mean value for the similarity of CD169 to F4/80 was 1.7 ± 0.2 (242 EBIs from four biological samples), whereas vascular cell adhesion molecule-1 (VCAM-1), which had a high correlation with F4/80 expression, has mean value of 2.2 ± 0.6 (811 EBIs, 10 biological samples) and CD11b, which was very dissimilar from F4/80, gives a mean value of 0.82 ± 0.4 (484 EBIs, 6 biological samples). Distributions shown are from representative experiments.

CD169^+^ macrophages in the bone marrow have been shown to promote retention of HSCs and erythroid progenitors (26, 27) and are presumed to be involved in EBIs. We tested a number of CD169 antibodies (3D6.112, REA197, and MOMA-1), which performed as reported in traditional flow cytometry (Figure 1), but gave inconsistent results in IFC and immunofluorescence microscopy. The best performing anti-CD169 for IFC and immunofluorescence was the REA197 clone (Miltenyi Biotec Inc.), but this also gave strong staining of the FL macrophages which are reported to lack CD169 (19, 28) so we restricted our analysis to the widely used (at least for flow cytometry) 3D6.112 clone. Though some EBI macrophages with clear CD169 staining were observed, many had little or no CD169 detectable (Figure 5A). Moreover, when CD169 was detectable on the EBI macrophages, the pattern was irregular rather than continuous membranous staining and, therefore, difficult to compare with the F4/80 signal. In order to compare the intermittent CD169 staining with the F4/80 expression on the central macrophage, a threshold mean pixel intensity was set to determine CD169 positivity and the similarity feature, a modification of Pearson’s correlation coefficient, was used to objectively determine if the CD169 staining was consistent with the F4/80. At least 90% of F4/80^+^ EBI macrophages were positive by this measure and the mean similarity score indicated that the staining, although heterogeneous, is more similar to F4/80 than not. For reference, this can be compared to the high similarity of VCAM-1 to F4/80, in contrast to CD11b and F4/80, which are not co-expressed (Figure 5B).

#### CD163 in Rat BM EBI Macrophages

**Figure 6:**
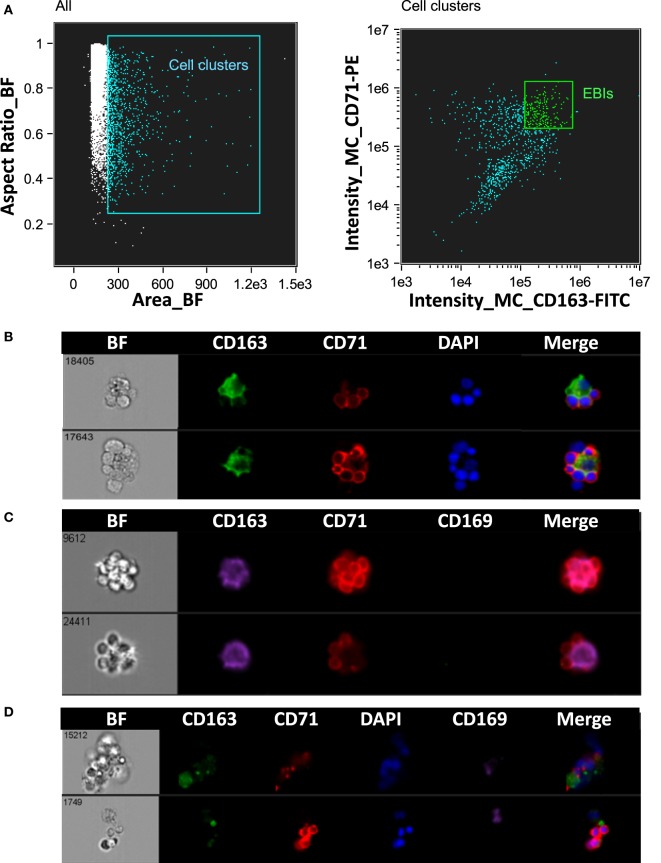
Analysis of erythroblastic islands (EBIs) from rat bone marrow by IFC. **(A)** Left panel: Multiplets (cell clusters) characterized by their large area in brightfield (BF) are gated. Right panel: The cell clusters are then plotted as per intensity of CD163 and of CD71 stain. The gate containing clusters high in fluorescence intensity for both CD163 and CD71 is enriched in EBIs (EBI gate). Manual inspection of the cell clusters in the EBI gate allows confirmation of the events that have clearly the structure of an EBI. **(B,C)** Examples of EBIs (within the EBI gate) characterized by a central CD163^+^/CD169^−^ macrophage surrounded by CD71^+^ erythroblasts. **(D)** Examples of cell clusters which although within the EBI gate (double positive for CD163 and CD71) do not have the classic appearance of an intact EBI. These events would have been included in the analysis by “blind” flow cytometry, potentially leading to misguided conclusions about EBI macrophage markers.

**Figure 7:**
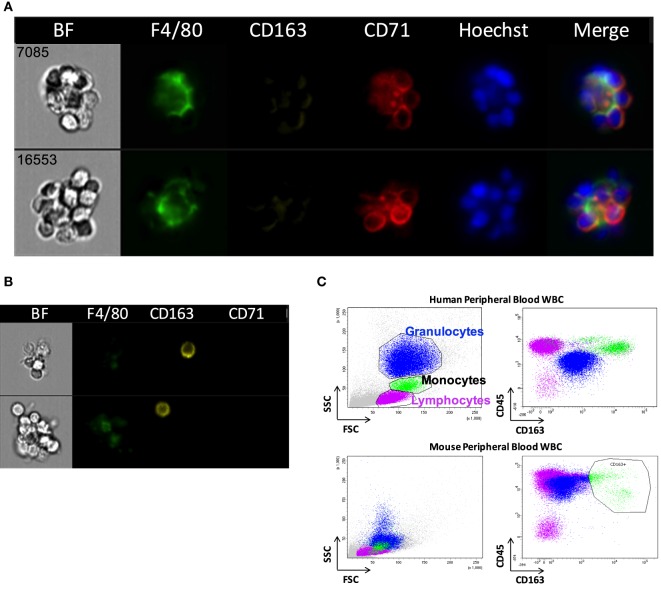
CD163 is poorly detectable in mouse erythroblastic island (EBI) macrophages by imaging flow cytometry. **(A)** Antibodies against CD163 did not reliably stain the mouse EBI macrophages. **(B)** There were cells at the same experiment, single or present in clusters, which were stained as CD163^+^. **(C)** Flow cytometric analysis of unfixed murine peripheral WBCs showed that the antibody used labels mouse peripheral blood cells as well as it labels human monocytes.

Erythroblastic islands were originally described in rat BM. We tested our method and IFC analysis strategy to prepare and visualize EBIs from BM isolated from Sprague-Dawley rats (Figure 6A). The central macrophage was consistently positive for CD163; CD169 was not detectable with clone ED3 (Figures 6B,C). Although the EBIs were enriched (20–44%) in the cell clusters within the CD71/CD163 double positive gate (Figure 6A), we could also identify clusters without the classic appearance of an island (Figure 6D) that had to be manually excluded for further analysis. CD163 has been identified as a membrane protein on rat and human BM macrophages and has been shown to function as an adhesion receptor for erythroblasts as well as a scavenger for hemoglobin–haptoglobin (Hb–Hp) complexes, contributing to the clearance of free hemoglobin (20). Interestingly, CD163 was not significantly detectable on murine BM EBI macrophages (Figure 7A), while it was easily detected by IFC on other cells present in the same BM preparation (Figure 7B) as well as by flow cytometry in a population of peripheral blood cells, indicating that the antibody does react with murine cells as well as it does with human monocytes (Figure 7C).

## Discussion

Macrophages are a heterogeneous population with wide distribution in different tissues and variable functions that range from integral participation in the immune defenses to their trophic role as stromal cells, supporting tissue homeostasis and regeneration (29). They even have variable origin with some of the tissue resident macrophages, including the resident BM macrophages (RBMMs), originating from the erythro-myeloid progenitors of the yolk sac and not from the HSC-derived myelo-monocytic lineage (30, 31). The RBMMs in the BM spread cellular processes to create an extended meshwork forming clusters with myeloid and erythroid cells (23). The clusters composed of erythroblasts surrounding a central macrophage were first observed almost 60 years ago as EBIs and have been proven over the years to be the erythropoietic niches, optimizing erythroblast proliferation, differentiation, survival, and enucleation (10, 14, 15).

We have evaluated here cell surface markers that have been used to define and study the murine EBI macrophages (25, 26, 32). The interactions of erythroblasts with the EBI macrophages had been described early on as either divalent cation dependent, mediated by a putative erythroblast receptor (EbR), or cation-independent (33). The cation-independent receptor was identified on RBMMs as a lectin-like agglutinin that could avidly bind non-opsonized sheep erythrocytes, and therefore, it was named initially SER (sheep erythrocyte receptor) (34). SER is now known as CD169, and it has been shown to be present in certain sub-populations of stromal tissue macrophages, especially those in lymph nodes, BM, liver, and SP, while it is low or undetectable on blood and BM monocytes and on macrophages isolated from thymus, peritoneal or pleural cavity, and bronchoalveolar spaces (34, 35). The divalent cation-dependent EbR was shown to be the dominant erythroblast adhesion receptor(s) on mouse bone marrow stromal macrophages (19) and was soon identified as the VCAM- (22) binding to α_4_β_1_ integrin on erythroblast membrane (36). The α_v_ integrin was also identified as a stromal macrophage receptor binding the erythroblast ICAM-4 and contributing an additional cation-dependent, integrin-mediated adhesive interaction (37).

None of these EBI macrophage markers is specific enough to be used as a single defining characteristic of this macrophage sub-population. Combinations of F4/80, VCAM-1, CD169, and CD11b have been considered a more reliable alternative (25, 26, 32). We used IFC to evaluate EBIs isolated from murine BM, stress-erythropoietic SP, and FL. IFC gives the advantage of high-throughput data analysis combined with the visualization of immunofluorescent staining for each event. With this technique, we were able to examine the phenotype and characteristics of EBIs and their central macrophages and identify morphological and immunophenotypic differences between different hematopoietic tissues in the mouse. We were also able to evaluate EBIs from rat BM, opening the possibility to investigate similarities and differences between species (24).

Our data demonstrated a variable degree of expression of F4/80, VCAM-1, and CD169, in the EBI macrophages between hematopoietic tissues and even within the same tissue, indicating that these markers are not only non-specific, but they also show a significant heterogeneity within this macrophage sub-population. A total of 5–20% of morphologically defined EBIs in the murine BM had a central macrophage without appreciable F4/80 positivity, in agreement with Crocker and Gordon who observed strong F4/80 staining in only 70% of the clusters they analyzed from murine BM (23). We found these F4/80^−^ central macrophages to express VCAM-1. The EBIs with F4/80^−^;VCAM-1^+^ macrophages were significantly increased in conditions of stress erythropoiesis: in BM and SP of anemic mice after phlebotomy, as well as in FLs, where erythropoiesis needs to meet the exceptionally fast rate of growth in embryonic life. This variation in the cell surface markers of EBI macrophages likely signifies a deeper biological difference between the erythropoietic niches under homeostatic and stress erythropoiesis. Along with distinct erythroid progenitors and cytokine signaling pathways (5), stress erythropoiesis may also utilize nurse macrophages of different origin and with special characteristics.

We also found wide heterogeneity of CD169 staining on the BM EBI macrophages by IFC. CD169 is a sialoadhesin that acts as a receptor for sialylated glycoconjugates and plays multiple and discrete roles in the hematopoietic, immune, and nervous systems, via adhesive and signaling interactions (38). A significant heterogeneity of CD169 distribution on the RBMMs was also demonstrated in the past, when this marker was first identified as SER (34). Crocker and Gordon showed by immunofluorescence and immunoelectron microscopy in mouse BM that SER is faintly stained at the border between macrophage and erythroblasts, whereas it is highly concentrated at the contact zones between macrophages and immature granulocytes and eosinophils (39). Of note, the same team demonstrated that binding of erythroblasts to FL macrophages is completely resistant to specific monoclonal antibody inhibitors of sialoadhesin and CD169 is not detectable on FL macrophages (19, 28). Thus, our difficulty imaging this receptor consistently in the EBI macrophages is likely due to its truly variable expression in this macrophage sub-population rather than to technical issues with anti-CD169 antibodies. This heterogeneity may also reflect heterogeneity in function of the corresponding EBI macrophages in erythropoiesis.

Most notable is our finding that the central macrophages in murine EBIs lack appreciable CD11b (Mac-1) expression in agreement with early studies in human and mouse BM stromal macrophages (13, 23). Therefore, this antigen is not an optimal marker to isolate or study EBI macrophages. Given that our studies by IFC showed the majority of BM EBIs to have associated CD11b^+^ cells, the CD71^+^;F4/80^+^ clusters would appear CD11b^+^ in a flow cytometer; therefore, use of this marker in a blind gating strategy may still yield a population of EBIs (25). However, our data show that CD11b is not on the F4/80^+^ macrophages themselves but on non-erythroid, non-macrophage cells associated with the islands. This finding is consistent with previous descriptions of BM cell clusters. Crocker and Gordon reported that 30% of BM clusters contained both erythroid and myeloid cells and immature myeloids were enriched as much as erythroid cells when clusters were purified by sedimentation (23). Moreover, CD169 is a receptor for granulocytes and eosinophils and is most highly concentrated at the contact zones between macrophages and these cells (39). It is intriguing to consider that myeloid cells may affect erythropoiesis via direct intercellular interactions within the erythropoietic niche, but further studies, including transcriptomics and IFC evaluation for other membrane markers of the granulocytic lineage, are needed in order to determine the identity and biological significance of these associated cells and their contribution(s) to the function and microenvironment of the EBIs.

We were also able to image EBIs from the rat BM. The central macrophage in these EBIs is characterized by the presence of CD163, a heme scavenger, which is however absent in mouse EBI macrophages, possibly implying a difference between species in erythropoiesis regulation. EBIs were first identified in the rat (10). Most of the studies in the field of erythropoiesis have used so far *in vitro* models of human stem and progenitor cells induced to differentiation toward red blood cells or *in vivo* mouse models because most of the techniques and reagents (e.g., knockout/knockdown technologies, antibodies) were restricted until recently to these animals. However, rat erythropoiesis is a more faithful model of human erythropoiesis, since unlike the mouse, stress erythropoiesis in the rat occurs mostly in the BM, similar to humans. With the recent development of CRISPR-Cas9, one may envision the rat as potential model for normal and disordered erythropoiesis (40).

Imaging flow cytometry has allowed us a better understanding of morphologic and phenotypic characteristics of the EBIs and their constituent cells and a clear demonstration of the heterogeneity of the EBI macrophage sub-population, which is not surprising based on the diversity and plasticity of the macrophages in general. Single-cell RNA-seq of macrophages isolated from EBIs may be better suited to clarify their heterogeneity and identify common intercellular interactions and signaling pathways assisting erythropoiesis as well as differences that may play a role in conditions of stress or pathologic erythropoiesis.

## Ethics Statement

This study was carried out in accordance with the recommendations from the Guide for the Care and Use of Laboratory Animals. The protocol was approved by the Institutional Animal Care and Use Committees of Cincinnati Children’s Hospital Medical Center, Cincinnati, OH, USA, and the Feinstein Institute for Medical Research, Manhasset, NY, USA.

## Author Contributions

KS, JP, LB, and TK designed and performed research, analyzed data and wrote the manuscript. RF and JH performed research and analyzed data. GH and NM contributed valuable reagents and instrumental suggestions on research design, data analysis, and writing of the manuscript.

## Conflict of Interest Statement

The authors declare that the research was conducted in the absence of any commercial or financial relationships that could be construed as a potential conflict of interest.

## Footnotes

^1^Based on calculations by Palis (1), considering that adult human blood normally contains approximately 5 × 106 RBCs/µL and the blood volume of a 70 kg person is approximately 5 L, the total circulating RBCs are 2.5 × 1013. With the lifespan of human RBCs being approximately 115 days (2), we replace on average 1/115th of our red cells every day, or 2.2 × 1011 RBC/day, which amounts to 2.5 million RBCs/sec. Similarly, adult mouse blood normally contains approximately 10 × 106 RBCs/µL and the blood volume of a 30 g adult mouse is approximately 2.4 mL, which amounts to 2.4 × 1010 total circulating RBCs. Since the lifespan of mouse RBCs is approximately 40 days (3) they replace on average 1/40th of their red cells every day, or 0.6 × 109 RBC/day, which amounts to ~7,000 RBCs/s.
